# Combined near-infrared excited SEHRS and SERS spectra of pH sensors using silver nanostructures†

**DOI:** 10.1039/c5cp03844h

**Published:** 2015-08-26

**Authors:** Marina Gühlke, Zsuzsanna Heiner, Janina Kneipp

**Affiliations:** a Department of Chemistry, Humboldt-Universität zu Berlin Brook-Taylor-Str. 2 12489 Berlin Germany janina.kneipp@chemie.hu-berlin.de; b School of Analytical Sciences Adlershof SALSA, Humboldt-Universität zu Berlin Zum Großen Windkanal 6 12489 Berlin Germany

## Abstract

Surface-enhanced hyper-Raman scattering (SEHRS) and surface-enhanced Raman scattering (SERS) of *para*-mercaptobenzoic acid (*p*MBA) were studied with an excitation wavelength of 1064 nm, using different silver nanostructures as substrates for both SEHRS and SERS. The spectra acquired for different pH values between pH 2 and pH 12 were compared with SERS data obtained from the identical samples at 532 nm excitation. Comparison of the ratios of the enhancement factors from SEHRS and SERS experiments with those from calculations using plasmonic absorbance spectra suggests that the difference between total surface-enhancement factors of SEHRS and SERS for *p*MBA is mainly explained by a difference between the electromagnetic contributions for linear and non-linear SERS. SERS and SEHRS spectra obtained at near-infrared (NIR) excitation indicate an overall reduction of enhancement by a factor of 2–3 at very low and very high pH, compared to neutral pH. Our data provide evidence that different molecular vibrations and/or different adsorption species are probed in SERS and SEHRS, and that SEHRS is very sensitive to slight changes in the *p*MBA–nanostructure interactions. We conclude that the combination of SEHRS and SERS using NIR excitation is more powerful for micro-environmental pH sensing than one-photon spectra excited in the visible range alone.

## Abstract 2

Combined surface-enhanced Raman scattering (SERS) and surface-enhanced hyper-Raman scattering (SEHRS) of a pH sensor, consisting of silver nanostructures and *para*-mercaptobenzoic acid and operating with near-IR excitation, is studied.

## Graphical abstract

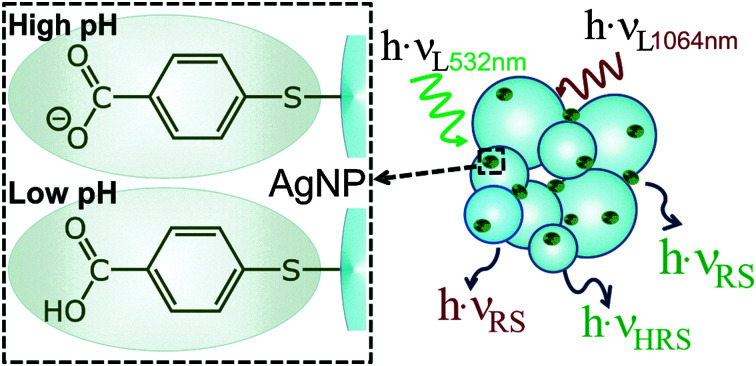

## Introduction

Vibrational spectroscopies, such as Raman and infrared spectroscopies, can be employed to characterize the structure, composition, and interaction of molecules and materials in a variety of analytical applications. Especially the combination of the one-photon excited linear Raman scattering and the two-photon excited non-linear hyper-Raman scattering, which occurs near the second harmonic of the excitation wavelength, can give a lot of structural information, because hyper-Raman scattering can offer information complementary to that from Raman spectroscopy and has some advantages over Raman and IR spectroscopies due to its different selection rules. The Raman-active vibrational modes of a centrosymmetric molecule are hyper-Raman-forbidden, and those inactive in both IR and Raman can be active in hyper-Raman scattering.^1^ In the presence of metal nanostructures, both one- and two-photon excited Raman scattering can profit from surface-enhancement. Surface-enhanced Raman scattering (SERS) allows us to capture data about the molecular structure and composition with high signal intensities in the strongly confined local electromagnetic fields of plasmonic nanostructures.^2,3^ In surface-enhanced hyper-Raman scattering, the inherently low two-photon cross-sections of the nonlinear hyper-Raman scattering can be overcome and increase to an order of 10^−46^–10^−45^ cm^4^ s.^4^

In the excitation range between 1000 nm and 1300 nm, mostly Fourier-transform (FT) techniques have been used in Raman spectroscopy; only in the past few years, technological advances allowed a more widespread use of the dispersive technique because of the revolution of non-silicon-based detectors.^5,6^ Thus, a direct comparison of Raman and hyper-Raman scattering, both excited at near infrared (NIR) wavelengths, but occuring at NIR and visible wavelengths, respectively, is possible. Nevertheless, in dispersive Raman spectroscopy at near infrared (NIR) wavelengths, the low Raman scattering intensities and detector sensitivities require long acquisition times. This can be improved by SERS, which was observed for NIR excitation first in FT-Raman on electrodes^7–9^ as well as on silver nanoparticles.^10,11^ Compared with its SERS spectrum, the SEHRS spectrum of a centrosymmetric molecule can contain new vibrational bands and display significant relative intensity differences because of the surface effect.^12^ In contrast, the two-photon excited spectrum of a non-centrosymmetric molecule largely resembles its SERS spectrum.^4,13,14^ Since the two processes give different insights into molecular symmetry, the combination of one- and two-photon NIR-excited SERS is a comprehensive way to investigate different molecules and molecule–nanostructure interactions.

An example of a molecule displaying qualitatively different SERS and SEHRS spectra is *para*-mercaptobenzoic acid (*p*MBA)^15^ which, similar to other organothiols,^16^ can strongly attach to silver nanostructures *via* its thiol group.^17,18^ Concentration-dependent changes of the orientation of the molecules on the silver surface have been monitored using the SERS spectra.^19^ Furthermore, in these spectra, the protonation and deprotonation of the carboxylate group in different pH environments can be observed,^19^ therefore the *p*MBA molecule can be used as a pH nanosensor, *e.g.*, in the endosomal system of live cells.^15,18,20^*p*MBA-based SERS pH sensors typically enable monitoring of a pH range of approximately pH 3.5 to pH 9 on silver and gold nanostructures.^15,21,22^ A first study employing two-photon excitation with *p*MBA on citrate-stabilized silver nanoparticles has suggested to use it for other pH ranges than excitation in the one-photon regime, specifically, the pH range of a SEHRS sensor could be extended to pH 2 in the acidic range.^15^

Here we discuss SEHRS spectra of *p*MBA acquired between pH 2 and 12 under non-resonant conditions in one combined microspectroscopic setup, together with the visible and near-infrared excited SERS spectra. Specifically, we report both SERS and SEHRS data obtained at an excitation wavelength of 1064 nm, and we show that this combination is more powerful for the determination of micro-environmental pH (*e.g.*, in biomaterials) and for the characterization of the sensor than one-photon spectra excited in the visible range at 532 nm alone. To better understand possible origins of the high pH sensitivity of NIR-excited SEHRS and SERS we have used several kinds of silver nanostructures.

## Materials and methods

### Chemicals

Silver nitrate (99.9999%), hydroxylamine hydrochloride (99%), sodium hydroxide (p.a.) and *para*-mercaptobenzoic acid (*p*MBA) (99%) were purchased from Sigma-Aldrich. Trisodium citrate dihydrate (99%) was purchased from Th. Geyer, and sodium borohydride and hydrochloric acid were purchased from J. T. Baker. Phosphate buffers of different pH values with a phosphate concentration of 0.1 M were prepared according to Sörensen's protocol^23^ with potassium dihydrogen phosphate (p.a., Merck) and disodium hydrogen phosphate (99%, Sigma-Aldrich).

### Synthesis of the pH nanosensors/sample preparation

Silver nanoparticles (AgNPs) were produced by chemical reduction of silver nitrate according to four different protocols. For citrate reduced AgNPs,^24^ 5 mL of a 0.04 M sodium citrate solution were added to a boiling solution of 45 mg of silver nitrate in 245 mL of water. The mixture was kept boiling for 1 hour. For hydroxylamine reduced AgNPs,^25^ 17 mg of silver nitrate were dissolved in 10 mL of water and added to 90 mL of an aqueous solution containing 12 mg of sodium hydroxide and 11 mg of hydroxylamine hydrochloride. The mixture was stirred for 1 hour. For the production of NaBH_4_ reduced, citrate stabilized AgNPs, two different procedures were used: (I) in the first procedure,^26^ a solution of 4.3 mg of silver nitrate in 250 mL of water was cooled to 4 °C and 0.9 mmol sodium citrate were added. Afterwards 1.25 × 10^−3^ mmol NaBH_4_ were added from a freshly prepared concentrated aqueous solution. The resulting particles are further referred to as Ag (NaBH_4_/citrate I). (II) In the second procedure,^27^ 0.65 mL of a 0.03 mM sodium citrate solution were added at 0 °C to 6.5 mL of a 1 mM AgNO_3_ solution. Afterwards, 0.35 mL of a 0.1 M NaBH_4_ solution were added and the mixture was stirred at 0 °C for 30 minutes. The resulting particles are further referred to as Ag (NaBH_4_/citrate II).

To form the nanosensors, silver nanoaggregates were mixed with *p*MBA to a final *p*MBA concentration of 9 × 10^−7^ M. pH was adjusted by ten times diluting the nanosensor samples with 0.1 M phosphate buffer solutions. Extremely high and low pH values were achieved by the addition of sodium hydroxide and hydrochloric acid, respectively.

### Characterization of the nanosensors

Absorbance spectra were recorded on a Jasco UV/visible/NIR spectrophotometer.

Transmission electron microscopy (TEM) images were obtained on holey carbon Cu-grids using a TECNAI G^2^20 S-TWIN and a Jeol JEM 2200-FS electron microscope operating at 200 kV.

### Raman experiments

The surface-enhanced Raman and hyper-Raman spectra were obtained using an imaging spectrometer by microprobe sampling (10× objective). For detection, a 1200 lines per mm grating blazed at 550 nm with a liquid nitrogen cooled CCD detector and a 600 lines per mm grating blazed at 1000 nm with a liquid nitrogen cooled InGaAs detector were used in the visible and NIR spectral range, respectively. The liquid samples were placed in microcontainers, and the one- and two-photon scattering was collected in confocal and epi-illumination. One-photon excited SERS spectra were typically accumulated for 1 s in the visible and for 10 s in the NIR range with laser powers of 10 mW from a 532 nm CW laser and 500 mW from a 1064 nm mode-locked laser. Hyper-Raman excitation with 300 mW at 1064 nm was provided by a mode-locked laser producing 7 ps pulses at a 76 MHz repetition rate. SEHRS spectra were accumulated for 2–15 s. Spectral resolution was 3–6 cm^−1^ in the full spectral range.

### Estimation of cross-sections and enhancement from Raman experiments

The scattering power in SERS and SEHRS, *P*^SERS^ and *P*^SEHRS^, measured in counts per second, was estimated at 1075 cm^−1^ and 1069 cm^−1^ (ring breathing mode) by SERS and SEHRS, respectively, according to

1 *P*^SERS^ = *N*_0_*σ*^SERS^*n*_L_

2 *P*^SEHRS^ = *N*_0_*σ*^SEHRS^*n*_L_^2^

where *σ*^SERS^ and *σ*^SEHRS^ are the effective cross-sections in SERS and SEHRS, respectively.^4^*N*_0_ is the number of molecules contributing to the signal (determined from an estimated area per molecule of 0.2 nm^2^ (ref. 28) and the nanoparticle surface available in the focal volume), and *n*_L_ is the excitation intensity (in photons per cm^2^ per second) for SERS and SEHRS. Knowing the excitation intensities and compensating for scattering powers with the different sensitivities and quantum efficiencies of the two types of detectors, amounting to a factor of 73 in favor of the SERS signal, a comparison between SEHRS and SERS signals measured from the same sample allows us to estimate a ratio of effective cross-sections in one-photon SERS and two-photon SEHRS based on eqn (1) and (2). The experimental ratio between the SERS and SEHRS power (*P*^SERS^/*P*^SEHRS^) can be combined with the corresponding signal ratio (*P*^RS^/*P*^HRS^) between non-enhanced Raman and hyper-Raman scattering to obtain a ratio between surface-enhancement factors of Raman and hyper-Raman scattering (*G*^SERS^/*G*^SEHRS^). The ratio between *P*^HRS^ and *P*^RS^ is not known from our experiment, but it can be roughly estimated for a certain excitation intensity based on previous work to be ∼5 × 10^−6^.^29^*G*^SERS^/*G*^SEHRS^ is the ratio of the total enhancement including electromagnetic and chemical contributions.

### Estimation of enhancement from absorbance spectra

The absorbance spectra can be used to determine empirically the electromagnetic SERS and SEHRS enhancement factors, *G*^SERS^ and *G*^SEHRS^, that can be obtained with the different nanoparticles according to eqn (3)^30^ and eqn (4),^14^ respectively3
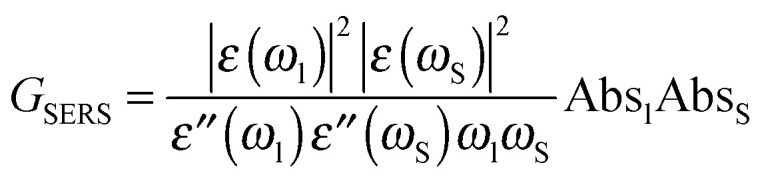
4
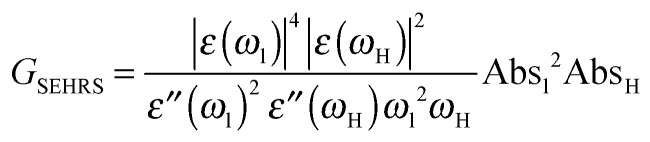
Here, Abs_l_, Abs_S_, and Abs_H_ are the absorbance of the sample at the laser, Stokes–Raman, and hyper-Raman wavelengths *λ*_L_ = *ω*_L_^−1^, *λ*_S_ = *ω*_S_^−1^, and *λ*_H_ = *ω*_H_^−1^, respectively. The Stokes wavenumber *ω*_S_ is *ω*_S_ = *ω*_L_*− ω*_m_, and the hyper-Raman wavenumber *ω*_H_ is *ω*_H_ = 2*ω*_L_ − *ω*_m_, where *ω*_m_ is the wavenumber of the molecular vibration. From Drude free electron theory, the complex dielectric constants of silver were determined^31^ at laser, Stokes, and hyper-Raman wavelengths as *ε̃*(*ω*_l_) = *ε*′(*ω*_l_) + *iε*′′(*ω*_l_), *ε̃*(*ω*_S_) = *ε*′(*ω*_S_) + *iε*′′(*ω*_S_), and *ε̃*(*ω*_H_) = *ε*′(*ω*_H_) + *iε*′′(*ω*_H_), respectively.

## Results and discussion

### Absorbance spectra and enhancement factors

**Fig. 1:**
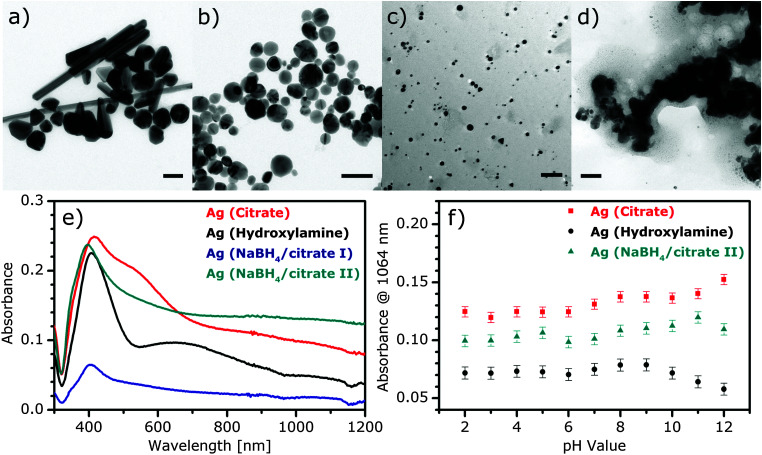
(a–d) TEM images of Ag nanoparticles (scale bars: 100 nm): citrate reduced/stabilized (a), hydroxylamine reduced (b), NaBH_4_ reduced, citrate stabilized I (c) and II (d). (e) Absorbance spectra of different Ag nanoparticles with *p*MBA (9 × 10^−7^ M) at pH 7. (f) Absorbance of citrate reduced/stabilized (red), hydroxylamine reduced (black) and NaBH_4_ reduced, citrate stabilized II (cyan) Ag nanoparticles as a function of pH value. Absorbance of various Ag NPs was monitored at 1064 nm excitation wavelength as a function of pH value. Error bars represent the standard deviation of absorbance values from three replicate measurements at pH 7.

Different types of silver nanostructures were synthesized by reduction with citrate, hydroxylamine, and sodium borohydride (following two different protocols), respectively. Transmission electron micrographs (TEM) of the different kinds of silver nanoparticles are shown in Fig. 1a–d. While citrate reduction gives relatively large particles with a significant amount of rod-like shapes (Fig. 1a), hydroxylamine reduction yields mostly spherical particles in a similar size (Fig. 1b). Reduction with borohydride, combined with citrate stabilization, either results in relatively small particles with an average diameter of 14 nm and a small fraction of triangular shaped nanoparticles (synthesis procedure I, Fig. 1c) or in large agglomerates (synthesis procedure II, Fig. 1d). Corresponding to the different size and shape of the nanoparticles, the nanosensors, consisting of these nanoparticles and *p*MBA, show variations in the position of the maximum absorbance and the shape of the spectra at neutral pH (Fig. 1e). The spectra of the sensors containing Ag (citrate) or Ag (hydroxylamine) are very reproducible for the pH range from pH 2 to 12 (ESI,† Fig. S1), indicating the stability of the nanoparticle solutions and their applicability for experiments with varying pH values. As Fig. 1f shows, the absorbance at 1064 nm of each nanoparticle solution is stable for different experiments conducted at acidic and neutral pH, while at basic pH slight variations in the extended plasmon band can be observed. We use this wavelength for the excitation of normal and hyper-Raman scattering and observe high enhancement in the SEHRS and SERS spectra despite the large gap between the excitation wavelength and the plasmon resonance maximum. This is in accord with previous theoretical and experimental findings, which describe high enhancement when frequency is changed from UV to near-IR wavelengths.^32–34^

The ratio of the electromagnetic enhancement of one-photon excited Raman scattering and hyper-Raman scattering can be estimated for a given absorbance spectrum of the sample and for a given excitation wavelength. Table 1 displays this ratio for the signal of the ring breathing vibration at around 1070 cm^−1^ at an excitation wavelength of 1064 nm at pH 7 (*cf.* spectra in Fig. 1e) resulting from eqn (3) and (4). In the case of Ag (hydroxylamine) nanoaggregates, we find a ratio of ∼10^−5^, while the ratio is ∼10^−4^ for Ag (citrate).

**Table 1:** Ratios of the cross-sections and enhancement factors of the different silver nanostructures at pH 7 as obtained in SERS and SEHRS experiments with an excitation wavelength of 1064 nm and determined from the absorbance data according to ref. 14 and 30

Ag nanoparticles at pH 7	Hydroxylamine	Citrate	NaBH_4_/citrate I
*σ* ^SERS^/*σ*^SEHRS^ [photons cm^−2^ s^−1^]	2 × 10^27^	3 × 10^27^	n.d.^a^
*G* ^SERS^/*G*^SEHRS^ from Raman experiments	9 × 10^−4^	8 × 10^−4^	n.d.^a^
*G* ^SERS^/*G*^SEHRS^ from EM field theory	3 × 10^−5^	9 × 10^−4^	6 × 10^−8^

Focal volume [μm^3^]			
In SERS	290	290	290
In SEHRS	37	37	37

Average particle diameter [nm]	42 ± 15	131 ± 29	14 ± 6

Number of molecules in the focal volume			
In SERS	2 × 10^5^	2 × 10^5^	2 × 10^5^
In SEHRS	2 × 10^4^	2 × 10^4^	2 × 10^4^

Number of particles in the focal volume			
In SERS	11	0.24	19
In SEHRS	1	0.03	2

a No SERS signal was obtained.

**Fig. 2:**
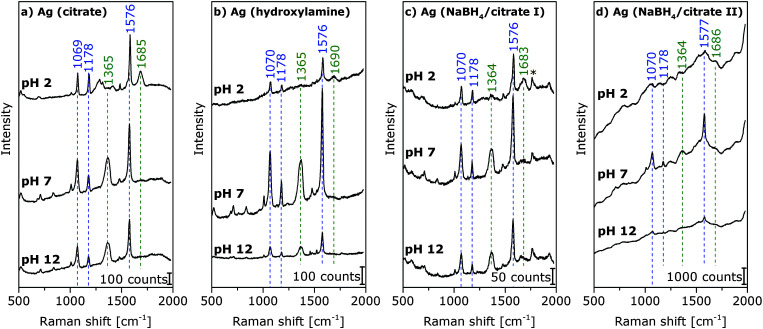
Surface-enhanced hyper-Raman spectra of *p*MBA in the local field of citrate reduced/stabilized (a) hydroxylamine reduced (b) and NaBH_4_ reduced, citrate stabilized I (c) and II (d) Ag nanoparticles at pH 2, 7, and 12. Excitation: 1064 nm, photon flux density: 2 × 10^25^ photons cm^−2^ s^−1^, acquisition time: 2 s (a and b), 15 s (c), and 10 s (d), and *p*MBA concentration: 9 × 10^−7^ M, averages of 30 spectra. The band marked with an asterisk in (c) also appears in the spectra of this nanoparticle type without *p*MBA.

Using SEHRS and SERS spectra obtained at pH 7, we have estimated the enhancement by the different silver nanostructures directly from Raman experiments. Example SEHRS and SERS spectra excited at 1064 nm are shown in Fig. 2 and 5, respectively. The different ratios of the cross-sections are also listed in Table 1. The enhancement factor ratios obtained from the Raman spectra agree with the empirical values from the absorbance spectra using eqn (3) and (4). This comparison shows that, in the case of *p*MBA, the difference between the total surface-enhancement factors of SEHRS and SERS can be explained by the difference between the corresponding electromagnetic contributions to the enhancement.

### pH-dependent SEHRS and SERS spectra

SEHRS spectra of *p*MBA were obtained at varying pH with silver nanostructures prepared according to four different protocols. The SEHRS spectra for pH 2, pH 7, and pH 12 are displayed as examples in Fig. 2. The Ag (NaBH_4_/citrate II) nanoparticles cause the formation of very large aggregates (see TEM in Fig. 1d), and a high background signal in all SEHRS experiments (Fig. 2d). In spite of their high stability (see also absorbance data in Fig. 1f), they are therefore not suitable for the construction of pH nanosensors. The spectra in Fig. 2a show the characteristic SEHRS signature of *p*MBA that was reported for citrate-reduced silver nanoaggregates previously.^15^ The spectra of *p*MBA obtained with Ag (hydroxylamine) (Fig. 2b) and Ag (NaBH_4_/citrate I) (Fig. 2c) are very similar for the corresponding pH values. They display differences in some relative band intensities compared to the spectra in Fig. 2a, due to different interactions of the *p*MBA molecules with the nanostructures. The high similarity of the spectra in Fig. 2b and c appears in spite of the high dissimilarity in surface coverage: while *p*MBA is present in excess on Ag (NaBH_4_/citrate I), leading to full surface coverage considering a space requirement given in ref. 28, the Ag (hydroxylamine) particles are covered to a much lesser extent (∼50%).

With increasing pH, in all spectra, the relative band intensities of vibrations associated with the carboxyl group (marked in green in Fig. 2) change relative to those of the aromatic ring (marked in blue in Fig. 2). Upon deprotonation, the intensity of the COO^−^ band at 1365 cm^−1^ increases, and the C=O stretching vibration at 1685 cm^−1^ decreases. This is in accord with the pH dependence of the one-photon SERS spectra excited at 532 nm (ESI,† Fig. S2).

Comparing the SEHRS spectra in Fig. 2 with the one-photon excited SERS spectra (ESI,† Fig. S2), we find in the SEHRS spectra smaller Raman shifts by 5–15 cm^−1^ for several bands (Fig. 2 and ESI,† Table S1). This is not specific for one nanoparticle type. These bands, which are seemingly shifted, can also indicate probing of different vibrations in SERS and SEHRS due to the different selection rules,^12^ and can be an indication that different adsorption species are probed in SERS and SEHRS. It is known that SEHRS is more sensitive to surface potential^13^ and to local surface environmental changes^12^ than SERS.

**Fig. 3:**
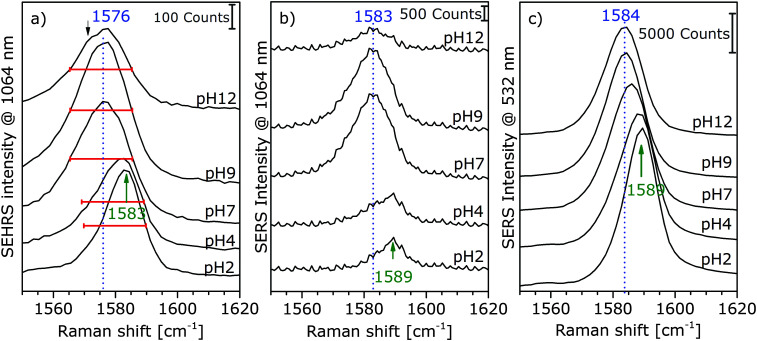
Extracts of SEHRS (a) and SERS spectra of *p*MBA with Ag (citrate) nanoparticles at an excitation wavelength of 1064 nm (b) and 532 nm (c). Experimental conditions as described in Fig. 2, 5, and Fig. S2 (ESI†), respectively.

For high pH values, new band components, such as asymmetric broadening and new shoulders, appear, which can also be an indication that different adsorption species are probed in SERS and SEHRS. Fig. 3a displays this for the example of the ring stretching vibration at 1585 cm^−1^ in the SEHRS spectra, while the band in the SERS data is only shifted and shows no second component (Fig. 3b and c). The appearance of a low-frequency shoulder in the SEHRS spectrum can be assigned to the non-totally symmetric ring stretching vibration, whereas the high-frequency component corresponds to the totally symmetric ring stretching vibration. The former was reported in SERS data as well, upon increasing charge transfer between the molecule and the silver surface,^35^ or when intermolecular interaction between the phenyl ring and a carboxylate group takes place.^36^ This indicates that at high pH, a larger fraction of the molecules must be adsorbed on the surface in a flat orientation and can be specifically probed by SEHRS (Fig. 3a). Apart from a small shift, this is not visible from the ring stretching band in the SERS spectra (Fig. 3b and c). In contrast, other bands in the SERS spectra support the same pH-dependent change in orientation found from the SEHRS data: for example, the low-frequency shoulder of the carboxylate stretching vibration at 1368 cm^−1^ (ESI,† Fig. S2) points to surface-bound carboxylate.^37^ The flat orientation is also evidenced by a higher intensity of bands from out-of-plane vibrations of the phenyl ring at 684 cm^−1^ and 710 cm^−1^ in both the SEHRS and SERS spectra (ESI,† Fig. S3) at neutral and basic pH.^19,35,38^ This is more pronounced in the SEHRS spectra, where both bands are clearly visible at neutral pH, and form one broad band with reduced intensity at acidic pH (ESI,† Table S1 and Fig. S3a). In the SERS spectra, only the broad band at 697 cm^−1^ and the band at 710 cm^−1^ are observed at acidic and neutral pH, respectively, and the C–H-deformation at 684 cm^−1^ does not appear (ESI,† Fig. S3b). These differences can be explained by the different selection rules that govern SEHRS, leading to preferred probing of those vibrations that are sensitive to pH-dependent surface interaction of the *p*MBA molecules. Similar effects were observed for other molecules.^12,13^

**Fig. 4:**
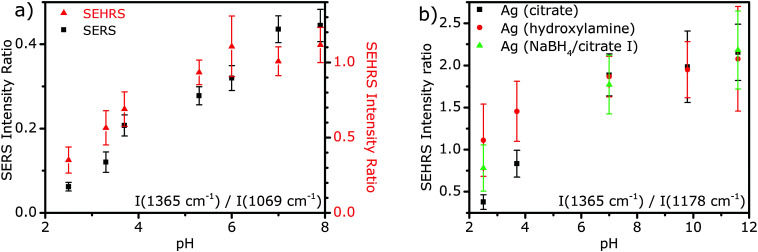
(a) Intensity ratios in the SERS spectra excited at 532 nm and in the SEHRS spectra excited at 1064 nm of *p*MBA with citrate reduced/stabilized NPs plotted as a function of pH for the bands at 1365 and 1069 cm^−1^ to demonstrate the operating range of SERS and SEHRS based pH-probes. (b) SEHRS intensity ratios in the spectra of *p*MBA with citrate (black), hydroxylamine (red) and NaBH_4_ (green) reduced AgNPs as a function of pH for the bands at 1365 and 1178 cm^−1^. One-photon excitation: 532 nm (CW), intensity: 3 × 10^5^ W cm^−2^, acquisition time: 1 s, and *p*MBA concentration: 9 × 10^−7^ M. Two-photon excitation: 1064 nm (7 ps/76 MHz), photon flux density: 2 × 10^25^ photons cm^−2^ s^−1^, acquisition time: 2 s, and *p*MBA concentration: 9 × 10^−7^ M. Positions of the bands are given according to the SEHRS spectra, for a comparison of band positions in SEHRS and SERS spectra see ESI,† Table S1. Intensity ratios are averaged over 30 spectra and error bars represent the corresponding standard deviations.

As proposed by us and others, pH sensing using *p*MBA SERS/SEHRS spectra relies on changes in relative intensities^15,20,21^ that are caused by both protonation and deprotonation of the molecule as well as by the resulting change in orientation at the nanoparticle surface.^19^ In Fig. 4a, the band ratio of the bands at 1365 cm^−1^ (COO^−^ stretching) and at 1069 cm^−1^ (ring breathing) from the SEHRS spectra is displayed as a function of pH (red triangles in Fig. 4a). Using this band ratio, acidic pH values can be clearly distinguished. Even though the sensors are stable at high pH (Fig. 1b and ESI,† Fig. S1), a nearly constant intensity of the carboxyl band at 1365 cm^−1^ in the *p*MBA spectrum for values above pH 8 prevents the discrimination of very similar, basic pH values. This is due to the uniform complete deprotonation of the carboxyl group above pH 8 (p*K*_a_ = 5 for the carboxyl group and p*K*_a_ = 5.8 for the thiol group).^39^ Therefore, the intensity ratio of pH-sensitive carboxyl and pH-insensitive aromatic bands in the SEHRS spectra is well-suited for the differentiation of acidic and neutral pH-values. From the comparison of the intensity ratios at different pH values for the different kinds of nanoparticles (Fig. 4b) we see that the sensitivity of a sensor made with citrate-stabilized silver nanostructures is highest, specifically in the acidic pH range. In Fig. 4a, we also compare the intensity ratios in the 1064 nm excited SEHRS spectra with the same intensity ratios in SERS spectra excited at 532 nm (black squares, for spectra please refer to ESI,† Fig. S2). The similar trend for the intensity ratio in the two types of spectra shows that, besides providing additional information on the interaction between the molecules and the metal surface, SEHRS spectra also contain the pH dependent spectral information that is obtained from the SERS spectra.

**Fig. 5:**
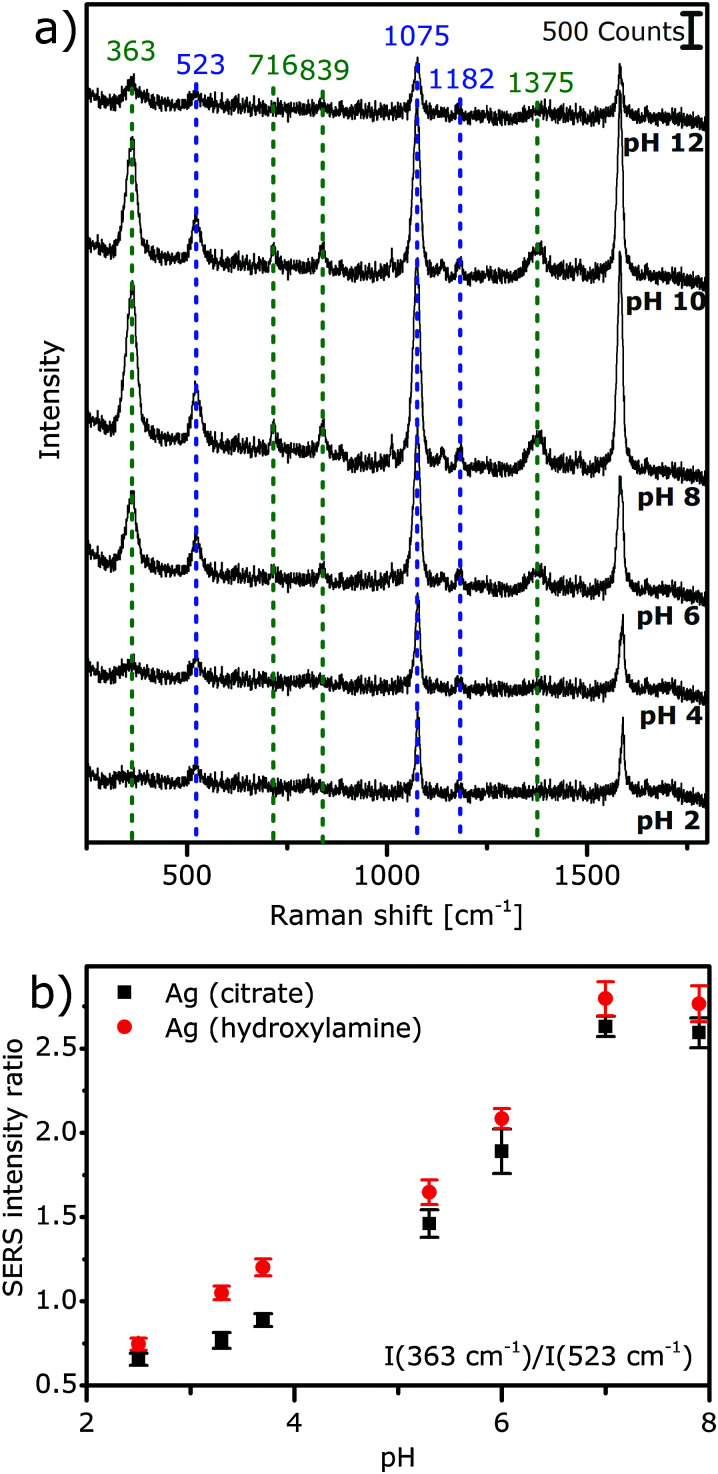
(a) One-photon excited surface enhanced Raman spectra of *p*MBA with citrate reduced/stabilized AgNPs with 1064 nm picosecond laser excitation in different pH environments. Photon flux density: 6 × 10^25^ photons cm^−2^ s^−1^, acquisition time: 10 s, and *p*MBA concentration: 9 × 10^−7^ M, averages of 30 spectra. (b) Intensity ratios in the spectra of *p*MBA with citrate (black) and hydroxylamine (red) reduced NPs as a function of pH for the bands at 363 and 523 cm^−1^. Intensity ratios are averaged over 30 spectra and error bars represent the corresponding standard deviations.

Considering the optimum pH range of the sensor in the acidic and neutral range, an application to biological systems, where the advantages of 2-photon excitation regarding material damage, spatial resolution, and penetration depth are obvious, using excitation at an infrared wavelength also for one-photon excitation is desirable. In Fig. 5a, we show SERS spectra that were excited with 1064 nm by the same laser as the SEHRS spectra and can be detected quasi-simultaneously in our microspectroscopic setup. Here, we utilize the intensity ratio of the pH-sensitive band at 363 cm^−1^ and of the pH-insensitive band at 523 cm^−1^ of a phenyl deformation vibration (see the band assignment in ESI,† Table S1), to discriminate pH values in the range between 2 and 7 (Fig. 5b). The band at 363 cm^−1^ can be assigned to deprotonated *p*MBA,^28^ particularly to a phenyl deformation combined with the C–S-stretching vibration^38,40^ and increases in intensity with pH becoming less acidic (Fig. 5a). Since its intensity is much stronger than that of the band at 710 cm^−1^ of the out-of-plane phenyl *γ*(C C C), that is used in the SEHRS spectra to probe the molecular orientation, it can serve as a very sensitive indicator for pH-induced changes in molecular orientation.

### pH-dependent enhancement of SEHRS and SERS

All nanoparticle types show a reduction of SEHRS enhancement by a factor of 2–3 at very low and very high pH, compared to neutral pH (Fig. 2a–d). The effect is strongest for the Ag (hydroxylamine) nanoparticles (Fig. 2b) and for Ag (NaBH_4_/citrate I) (Fig. 2c). Also, one-photon SERS excited at 1064 nm (Fig. 5a) shows similar behavior, *i.e.* an increase of the overall signal when the pH changes from acidic to neutral, and a decrease again at high pH. This effect is not observed in 532 nm excited SERS spectra with the same samples (ESI,† Fig. S2). Especially for the acidic pH values, the change in overall enhancement supports an influence of the surface chemistry, such as an altered interaction of the molecule with the metal nanoparticle surface, as was proposed previously,^19^ or with the stabilizing species.^41^ In particular, the stabilizing citrate and hydroxylamine/hydroxide contain functional groups that can be protonated or deprotonated, depending on the surrounding pH.

Due to the resulting changes in the nanoparticle surface, charge aggregation of the nanosensors, rather than severe Ag-nanostructure modifications that were observed as a function of the concentration of some organic molecules,^42^ could take place: for example, the negative surface charge of the silver particles decreases with decreasing pH by protonation, leading to increased aggregation and minor changes in gap sizes in the nanoaggregates and therefore to a lower electromagnetic enhancement at very acidic pH.^43,44^ This can remain undetected in the absorbance spectra if occurring only for a small fraction of the nanoparticles,^45^ but it could greatly change electromagnetic enhancement for some of the nanosensors.^46^

Apart from changes to the stabilizing species, a protonation of the thiolate could weaken the Ag–S-bond and thus change the interaction between *p*MBA itself and the silver surface at acidic pH. On the opposite side, at basic pH, where both, the stabilizing molecules and *p*MBA, are deprotonated, the repulsion between the negative charges of the analyte molecule and the nanoparticle surface is high,^43^ which alters the interaction between the metal surface and *p*MBA and thus leads to decreased enhancement. At neutral and slightly basic pH the two effects are balanced, and the interaction of the molecule with the metal surface leads to the highest enhancement.

## Conclusions

In conclusion, the combination of near-infrared excited SEHRS and SERS on the same sample, in one microspectroscopic setup along with the option to measure SERS using the second harmonic of the excitation laser, offers new possibilities for comparing one- and two-photon-excited SERS. This can provide deeper insight into the enhancement mechanism in linear and non-linear SERS and for the examination of interactions of molecules with different metal nanostructures. Here, we have investigated SERS and SEHRS of *p*MBA on silver nanostructures in a varying pH environment.

Our experiments identify advantages of SEHRS over SERS regarding improved vibrational spectroscopic selectivity and an extended pH-detection range as can be seen from the SEHRS spectra of *p*MBA at varying pH. In addition, as a two-photon excited spectroscopy, SEHRS benefits from a decreased probed volume.

Our studies suggest new possibilities regarding sensing applications by exploiting combined SEHRS/SERS measurements using NIR excitation. As we have shown, a combined SEHRS/SERS pH sensor which uses *p*MBA is most sensitive in the acidic and neutral pH ranges. This makes it especially useful for the examination of biological objects, which generally profits from near-infrared excitation. For future applications, combining nonlinear and linear NIR excitation in SERS, together with the tunable optical properties of plasmonic nanoparticles, will open up new possibilities for microscopic bio-sensing.

## Supplementary Material

CP-017-C5CP03844H-s001
