# Supplementary material for: Combined near-infrared excited SEHRS and SERS spectra of pH sensors using silver nanostructures
Source: Phys Chem Chem Phys. 2015 Aug 26;17(39):26093–100. doi: 10.1039/c5cp03844h (PMC11292597; doi:10.1039/c5cp03844h)
Supplement: CP-017-C5CP03844H-s001 [file CP-017-C5CP03844H-s001.pdf]

Electronic Supplementary Material (ESI) for Physical Chemistry Chemical Physics.
This journal is © the Owner Societies 2015

Combined near-infrared excited SEHRS and SERS spectra for pH sensors using silver nanostructures

Marina Gühlke[‡,a], *Zsuzsanna Heiner*[‡,a,b], *Janina Kneipp*[*,a,b]

[a] Department of Chemistry, Humboldt-Universität zu Berlin, Brook-Taylor-Str. 2, 12489 Berlin, Germany

[b] School of Analytical Sciences Adlershof SALSA, Humboldt-Universität zu Berlin, Zum Großen Windkanal 6, 12489 Berlin, Germany

Electronic Supplementary Information

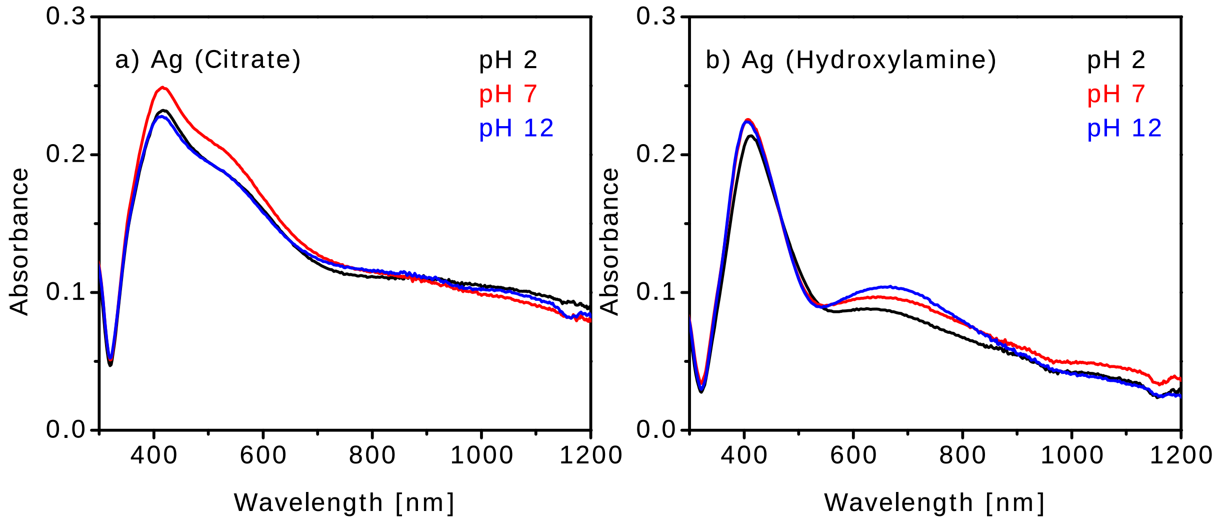

Fig. S1. Absorbance spectra of citrate reduced/stabilized (a) and hydroxylamine reduced (b) Ag nanoparticles with *p*MBA at different pH values.

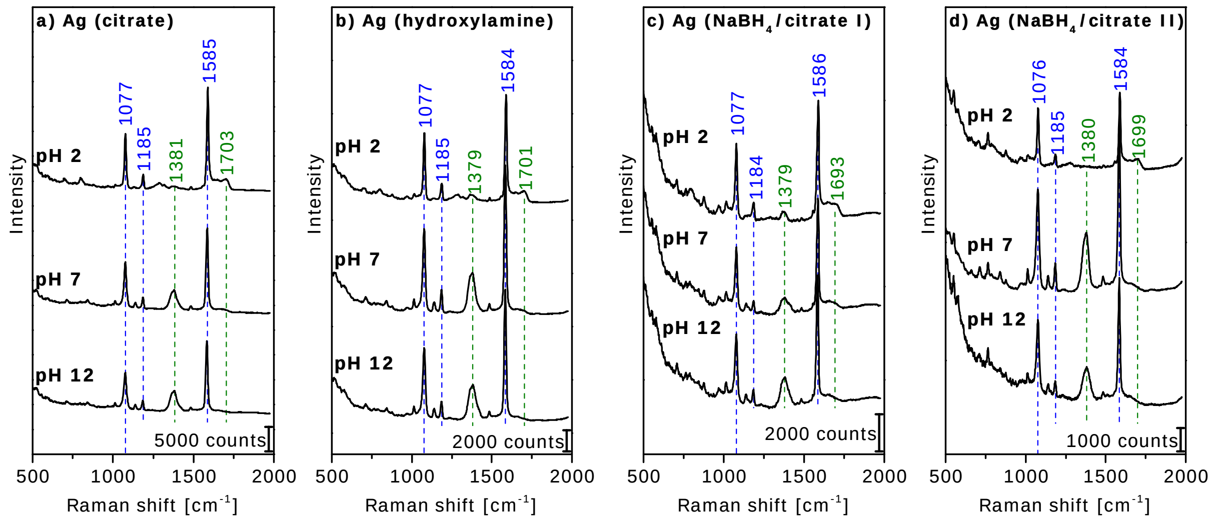

Fig. S2. SERS spectra of *p*MBA excited at 532 nm with citrate reduced/stabilized **(a)**, hydroxylamine reduced **(b)**, NaBH4 reduced, citrate stabilized I **(c)** and II **(d)** Ag nanoparticles. Excitation intensity: 3 x 10^5 W/cm^2, acquisition time: 1 s (a,b,d), 2 s (c), *p*MBA concentration: 9 x 10^{-7} M, averages of 30 spectra.

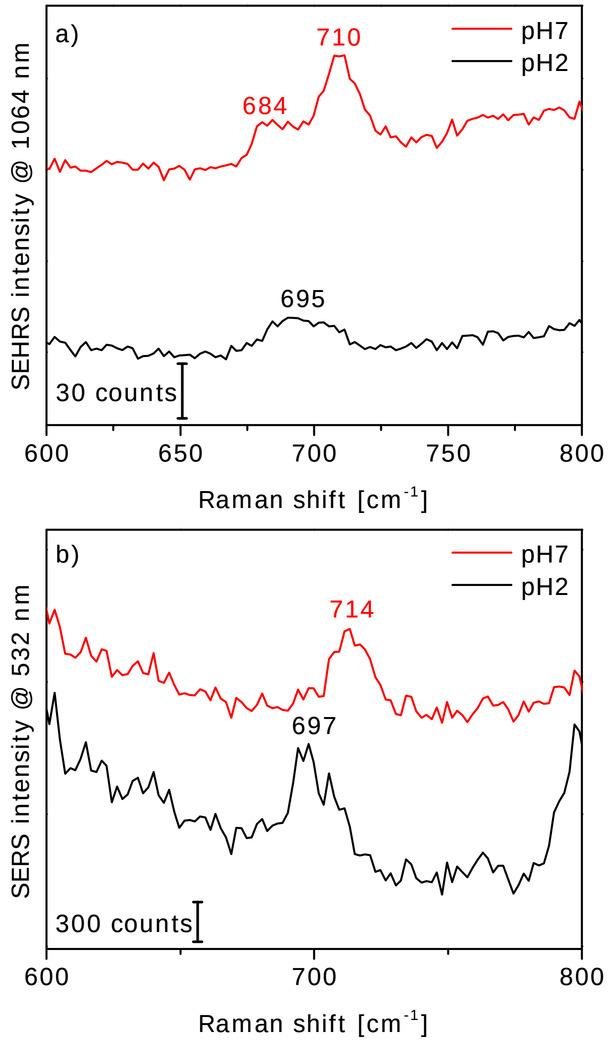

Fig. S3. Extracts of SEHRS **(a)** and SERS spectra **(b)** of *p*MBA with Ag (citrate) nanoparticles at an excitation wavelength of 1064 nm and 532 nm, respectively. Experimental conditions as described in Fig. 2 and S2, respectively.

Table S1. Band positions (cm^{-1}) in SEHRS and SERS spectra of *p*MBA with Ag (citrate) at pH 7 and band assignments.

SEHRS (1064 nm)	SERS (1064 nm)	SERS (532 nm)	Assignment	References
	363		phenyl deformation + C-S-stretching	1, 2
520	523		in-plane ring deformation	2, 3
684			C-H out-of-plane deformation	4
695*		697*	C-H out-of-plane deformation + out-of-plane γ(CCC)	
710	716	714	out-of-plane γ(CCC)	1, 4, 5
		800*	out-of-plane C-COOH	6
836	839	842	COO^- deformation	1, 5, 6
1009	1012	1013	ring deformation	7
1069	1075	1077	ring breathing	7
	1138	1139	C-COO^- stretching	7
1178	1182	1185	C-H-in-plane-bending	7
1365		1368 (shoulder)	COO^- stretching (COO^- surface-bound)	5, 8
	1375	1381	COO^- stretching (COO^- non-surface-bound)	5
1479		1485	C-H in-plane-bending	7
1576	1583	1585	ring stretching	7
1685*		1703*	C=O stretching of protonated carboxyl group	5

*band only observed at acidic pH

References

1 S. B. Lee, K. Kim and M. S. Kim, *J. Raman Spectrosc.*, 1991, **22**, 811-817.

2 R. A. Alvarez-Puebla, D. S. Dos Santos and R. F. Aroca, *Analyst*, 2004, **129**, 1251-1256.

3 A. S. L. Lee and Y. S. Li, *Spectrochim. Acta, Part A*, 1996, **52**, 173-184.

4 Y. Wang, W. Ji, H. M. Sui, Y. Kitahama, W. D. Ruan, Y. Ozaki and B. Zhao, *J. Phys. Chem. C*, 2014, **118**, 10191-10197.

5 A. Michota and J. Bukowska, *J. Raman Spectrosc.*, 2003, **34**, 21-25.

6 C. X. Ma and J. M. Harris, *Langmuir*, 2011, **27**, 3527-3533.

7 Y. Liu, H. Yuan, A. M. Fales and T. Vo-Dinh, *J. Raman Spectrosc.*, 2013, **44**, 980-986.

8 H. K. Park, S. B. Lee, K. Kim and M. S. Kim, *J. Phys. Chem.*, 1990, **94**, 7576-7580.
